# Supplementary material for: Childhood body size directly increases type 1 diabetes risk based on a lifecourse Mendelian randomization approach
Source: Nat Commun. 2022 Apr 28;13:2337. doi: 10.1038/s41467-022-29932-y (PMC9051135; doi:10.1038/s41467-022-29932-y)
Supplement: Supplementary file 1 — Supplementary Information [file 41467_2022_29932_MOESM1_ESM.pdf]

Supplementary Figures

Supplementary Figure 1:

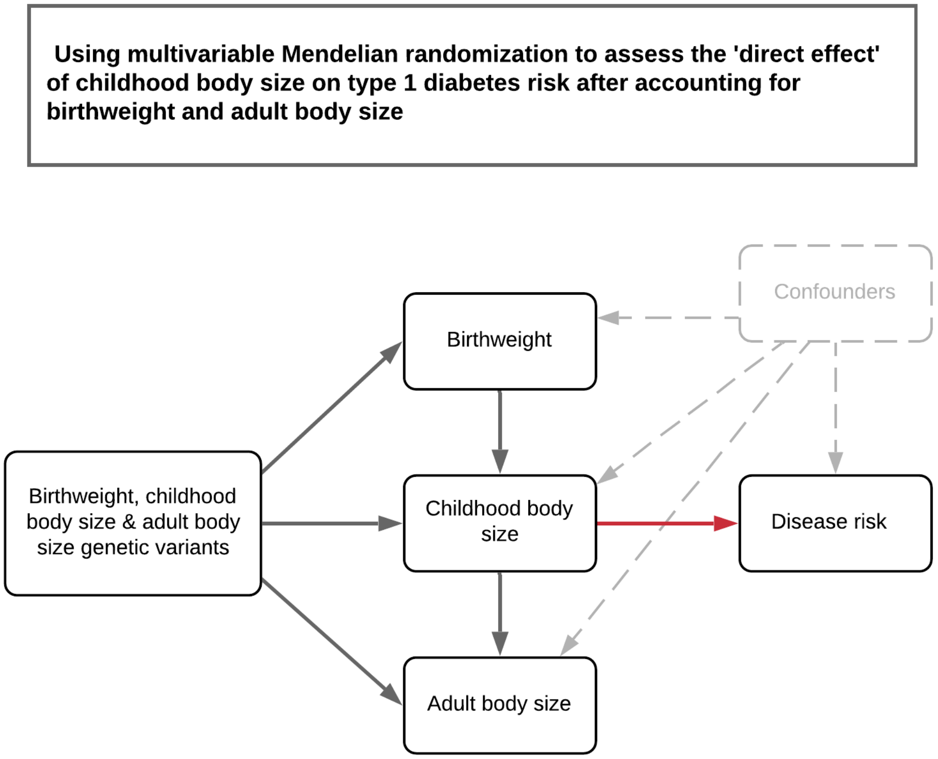

A schematic representation of the analysis undertaken in this study to assess the direct effect of childhood body after accounting for both birthweight and adult body size using multivariable Mendelian randomization. We emphasise that this analysis was undertaken to investigate whether birthweight was potentially responsible for the direct effect of childhood adiposity, as opposed to rigourously exploring whether parental effects influence type 1 diabetes risk.

Supplementary Figure 2:

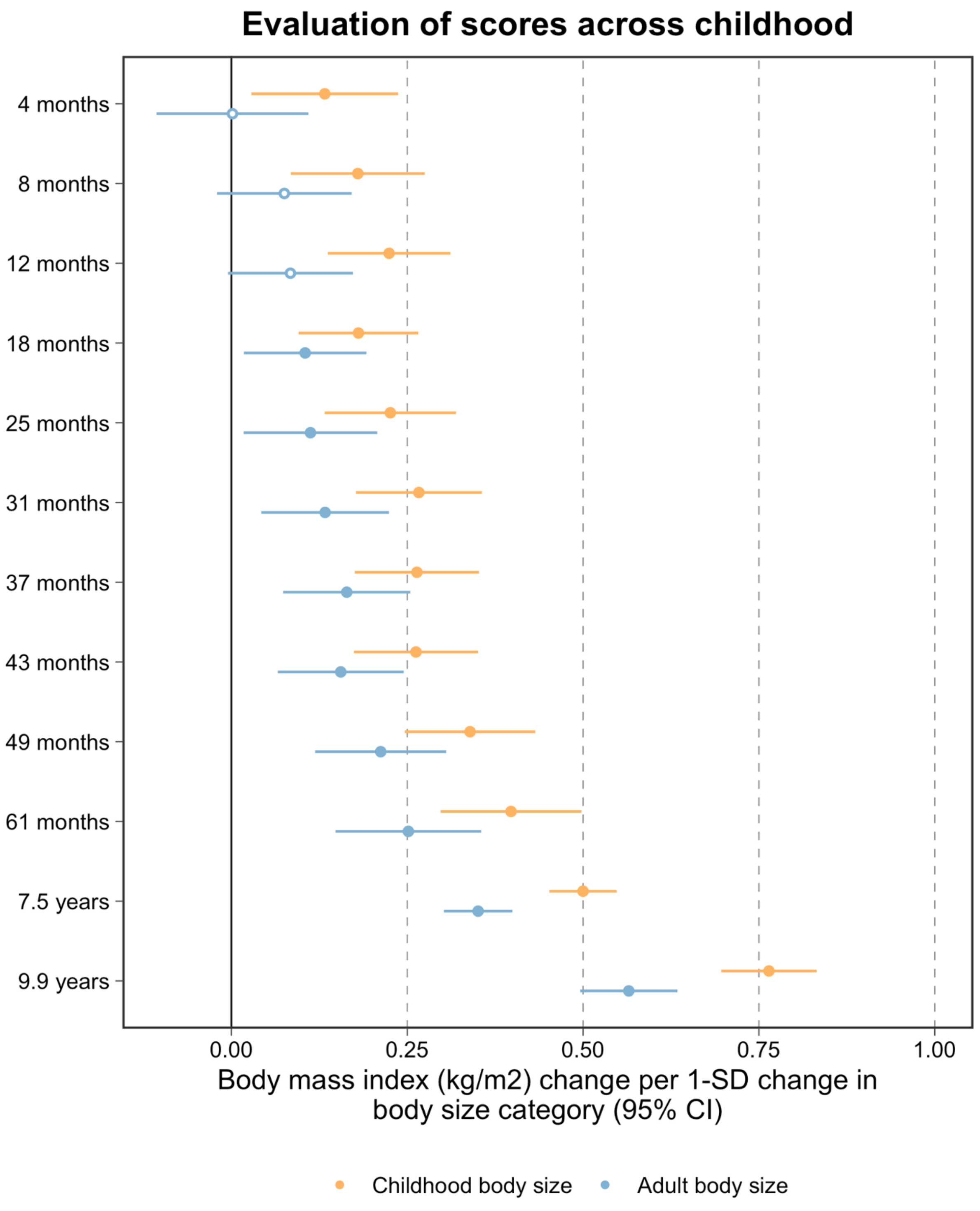

Linear regression results to evaluate the association between our childhood (yellow) and adult (blue) body size genetic instruments with measured body mass index at 12 separate timepoints in the Avon Longitudinal Study Parents and Children (ALSPAC). Effect estimates and sample sizes at each timepoint are reported in Supplementary Table 8.

Supplementary Figure 3:

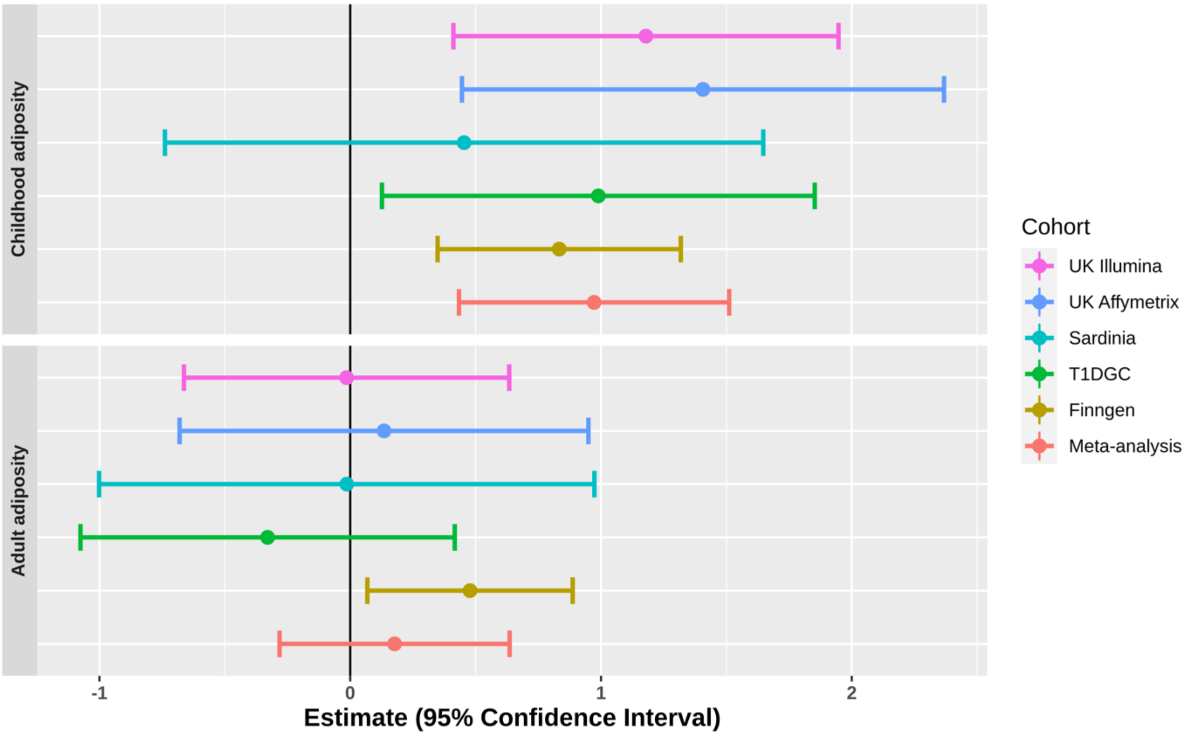

Multivariable Mendelian randomization analyses of childhood and adult body size (n=453,169) on type 1 diabetes risk undertaken on each contributing study to the large-scale meta-analysis used in this work (n=173,981). Estimates are based on the MR-Egger method and can be found in Supplementary Tables 13 and 14.

Supplementary Figure 4:

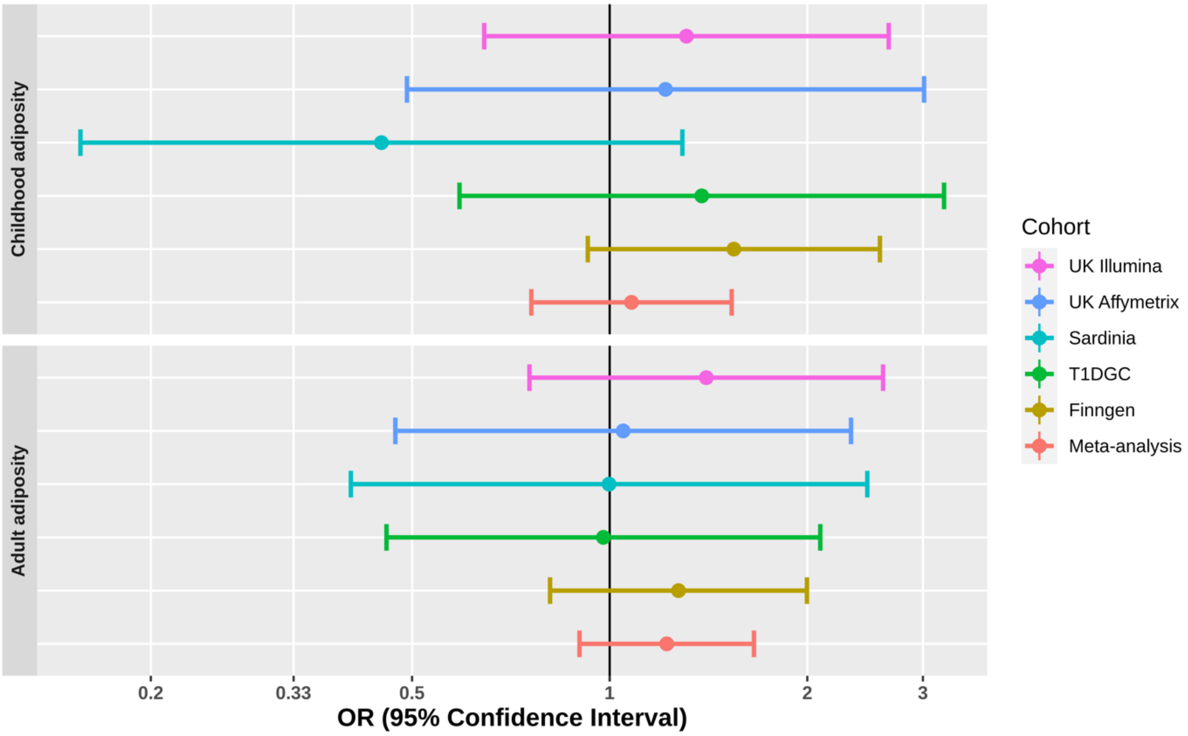

Multivariable Mendelian randomization analyses of childhood and adult body size (n=453,169) on type 1 diabetes risk undertaken on each contributing study to the large-scale meta-analysis used in this work (n=173,981). Estimates are based on the weighted median method and can be found in Supplementary Tables 13 and 14.
