# Supplementary material for: Childhood body size directly increases type 1 diabetes risk based on a lifecourse Mendelian randomization approach
Source: Nat Commun. 2022 Apr 28;13:2337. doi: 10.1038/s41467-022-29932-y (PMC9051135; doi:10.1038/s41467-022-29932-y)
Supplement: Supplementary file 3 — Description of Additional Supplementary Files [file 41467_2022_29932_MOESM3_ESM.pdf]

Description of Additional Supplementary Files

File Name: Supplementary Data 1
Description: Age at diagnosis information for the type 1 diabetes meta-analysis cohorts

File Name: Supplementary Data 2
Description: A summary of the genetic datasets used in this study

File Name: Supplementary Data 3
Description: Genetic variants associated with type 1 diabetes risk (from Crouch et al)

File Name: Supplementary Data 4
Description: Univariable Mendelian randomization analyses for childhood and adult body size onto type 1

File Name: Supplementary Data 5
Description: Univariable Mendelian randomization analyses for childhood and adult height onto type 1 diabetes risk

File Name: Supplementary Data 6
Description: Univariable Mendelian randomization analyses for childhood and adult body size onto type 1 diabetes risk using data from the T1D meta-analysis

File Name: Supplementary Data 7
Description: Univariable Mendelian randomization analyses to evaluate the effect of type 1 diabetes genetic liability on childhoood and adult body size using data from the T1D meta-analysis

File Name: Supplementary Data 8
Description: Associations between childhood and adult body size genetic scores with repeated measures of body mass index in the ALSPAC study

File Name: Supplementary Data 9
Description: Multivariable Mendelian randomization analyses for childhood and adult body size onto type 1 diabetes risk

File Name: Supplementary Data 10
Description: Multivariable Mendelian randomization analyses for childhood and adult body size onto type 1 diabetes risk using the MR-Egger method

File Name: Supplementary Data 11
Description: Multivariable Mendelian randomization analyses for childhood body size, adult body size and birthweight onto type 1 diabetes risk

File Name: Supplementary Data 12
Description: Univariable Mendelian randomization analyses for birthweight onto type 1 diabetes risk

File Name: Supplementary Data 13

Description: Univariable and multivariable Mendelian randomization analyses for childhood and adult body size onto type 1 diabetes risk using data from the T1D meta-analysis

File Name: Supplementary Data 14
Description: Multivariable Mendelian randomization analyses for childhood and adult body size onto type 1 diabetes risk using data from each T1D cohort individually

File Name: Supplementary Data 15
Description: Estimated change in prevalence of T1D with falling obesity

File Name: Supplementary Data 16
Description: Univariable and multivariable Mendelian randomization analyses for childhood and adult body size onto type 2 diabetes risk

File Name: Supplementary Data 17
Description: Univariable and multivariable Mendelian randomization analyses for childhood and adult body size onto 7 immune-associated endpoints using the IVW method
