# Supplementary material for: Childhood body size directly increases type 1 diabetes risk based on a lifecourse Mendelian randomization approach
Source: Nat Commun. 2022 Apr 28;13:2337. doi: 10.1038/s41467-022-29932-y (PMC9051135; doi:10.1038/s41467-022-29932-y)
Supplement: Supplementary file 5 — Reporting Summary [file 41467_2022_29932_MOESM5_ESM.pdf]

nature portfolio

Corresponding author(s): Tom Richardson

Last updated by author(s): Mar 14, 2022

Reporting Summary

Nature Portfolio wishes to improve the reproducibility of the work that we publish. This form provides structure for consistency and transparency in reporting. For further information on Nature Portfolio policies, see our Editorial Policies and the Editorial Policy Checklist.

Statistics

For all statistical analyses, confirm that the following items are present in the figure legend, table legend, main text, or Methods section.

n/a	Confirmed	
☐	☒	The exact sample size (*n*) for each experimental group/condition, given as a discrete number and unit of measurement
☐	☒	A statement on whether measurements were taken from distinct samples or whether the same sample was measured repeatedly
☐	☒	The statistical test(s) used AND whether they are one- or two-sided<br>*Only common tests should be described solely by name; describe more complex techniques in the Methods section.*
☐	☒	A description of all covariates tested
☐	☒	A description of any assumptions or corrections, such as tests of normality and adjustment for multiple comparisons
☐	☒	A full description of the statistical parameters including central tendency (e.g. means) or other basic estimates (e.g. regression coefficient) AND variation (e.g. standard deviation) or associated estimates of uncertainty (e.g. confidence intervals)
☐	☒	For null hypothesis testing, the test statistic (e.g. *F*, *t*, *r*) with confidence intervals, effect sizes, degrees of freedom and *P* value noted<br>*Give P values as exact values whenever suitable.*
☒	☐	For Bayesian analysis, information on the choice of priors and Markov chain Monte Carlo settings
☒	☐	For hierarchical and complex designs, identification of the appropriate level for tests and full reporting of outcomes
☒	☐	Estimates of effect sizes (e.g. Cohen's *d*, Pearson's *r*), indicating how they were calculated

Our web collection on statistics for biologists contains articles on many of the points above.

Software and code

Policy information about availability of computer code

Data collection	No software was used for data collection
Data analysis	Two-sample MR analyses were conducted using the TwoSampleMR package (version 0.5.5) in R (version 3.5.3). Multivariable MR analysis was conducted using both the MVMR (version 0.2.0).

For manuscripts utilizing custom algorithms or software that are central to the research but not yet described in published literature, software must be made available to editors and reviewers. We strongly encourage code deposition in a community repository (e.g. GitHub). See the Nature Portfolio guidelines for submitting code & software for further information.

Data

Policy information about availability of data

All manuscripts must include a data availability statement. This statement should provide the following information, where applicable:
- Accession codes, unique identifiers, or web links for publicly available datasets
- A description of any restrictions on data availability
- For clinical datasets or third party data, please ensure that the statement adheres to our policy

All individual level data analysed in this study can be accessed via an approved application to ALSPAC (http://www.bristol.ac.uk/alspac/researchers/access/). Summary statistics on type 1 and type 2 diabetes are publicly available from the studies as referenced in Supplementary Table 2. All other summary statistics analysed in this study can be accessed via the OpenGWAS (https://gwas.mrcieu.ac.uk/) and the FinnGen (https://www.finngen.fi/fi) resources.

Field-specific reporting

Please select the one below that is the best fit for your research. If you are not sure, read the appropriate sections before making your selection.

- [x] Life sciences
- [ ] Behavioural & social sciences
- [ ] Ecological, evolutionary & environmental sciences

For a reference copy of the document with all sections, see nature.com/documents/nr-reporting-summary-flat.pdf

Life sciences study design

All studies must disclose on these points even when the disclosure is negative.

Sample size	Our study analysed summary level data from published genome-wide association studies (GWAS) and therefore sample sizes were predetermined as described in their corresponding publications. All sample sizes are reported in Supplementary Table 2 along with their corresponding publications (including pubmed IDs 32376654, 28763444, 28566273, 29273806, 24390342 & 28067908). Sample sizes in ALSPAC consisted of all participants in this cohort with measures of body mass index (at each timepoint analysed), genome-wide genotyping, age and sex.
Data exclusions	Data on individuals from the ALSPAC cohort were only excluded if participants had withdrawn consent, were of non-European descent or if they had an older sibling already included in the dataset. Data exclusions from summary-level datasets varied according to their relevant publications listed in the Sample size section (alternatively these can be found in Supplementary Table 2).
Replication	Replication analyses were conducted using a larger GWAS meta-analysis than analysed in our discovery analysis, which strongly supported findings in this study.
Randomization	Our approach (Mendelian randomization) is based on the quasi randomization of genetic variants at conception.
Blinding	Our study using Mendelian randomization meant that blinding was not required.

Reporting for specific materials, systems and methods

We require information from authors about some types of materials, experimental systems and methods used in many studies. Here, indicate whether each material, system or method listed is relevant to your study. If you are not sure if a list item applies to your research, read the appropriate section before selecting a response.

Materials & experimental systems

n/a	Involved in the study	
☒	☐	Antibodies
☒	☐	Eukaryotic cell lines
☒	☐	Palaeontology and archaeology
☒	☐	Animals and other organisms
☐	☒	Human research participants
☒	☐	Clinical data
☒	☐	Dual use research of concern

Methods

n/a	Involved in the study	
☒	☐	ChIP-seq
☒	☐	Flow cytometry
☒	☐	MRI-based neuroimaging

Human research participants

Policy information about studies involving human research participants

Population characteristics	Characteristics of the studies analysed in this work are described in Supplementary Table 2. As described in our methods section, genome-wide association studies conducted in this study were adjusted for age, sex and genotyping chip in the model. ALSPAC analyses were adjusted for age and sex.
Recruitment	Summary-level data was primarily analysed in this study and therefore recruitment details can be found in their corresponding publications. Recruitment in the ALSPAC cohort is described in our methods section and is detailed previously (Boyd, A. et al. Cohort Profile: the 'children of the 90s'--the index offspring of the Avon Longitudinal Study of Parents and Children. Int J Epidemiol 42, 111-127, doi:10.1093/ije/dys064 (2013)). In brief, ALSPAC's eligible sample is defined as all pregnant women living in and around the city of Bristol (south-west UK) and due to deliver between April 1991 and December 1992. Women carrying a total of 20 248 pregnancies were deemed eligible.
Ethics oversight	Written informed consent was obtained for all study participants. Ethical approval for this study was obtained from the ALSPAC Ethics and Law Committee and the Local Research Ethics Committees. All individual participant data used from the UKB study had ethics approval from the Research Ethics Committee (REC; approval number: 11/NW/0382) and informed consent from all participants enrolled in UKB.

Note that full information on the approval of the study protocol must also be provided in the manuscript.
